# Complete Genome Sequences of Three *Salmonella enterica* subsp. *enterica* Serovar Saintpaul Isolates Associated with a 2013 Multistate Outbreak in the United States

**DOI:** 10.1128/genomeA.00456-17

**Published:** 2017-06-01

**Authors:** Kuan Yao, Tim Muruvanda, Marc W. Allard, Maria Hoffmann

**Affiliations:** a Division of Microbiology, Office of Regulatory Science, Center for Food Safety and Nutrition, U.S. Food and Drug Administration, College Park, Maryland, USA; b School of Systems Biology, George Mason University, Manassas, Virginia, USA

## Abstract

In 2013, a multistate outbreak of *Salmonella enterica* subsp. *enterica* serovar Saintpaul from cucumber caused 84 cases of salmonellosis in the United States. In this announcement, we report the complete genome sequences of three clinical *Salmonella* Saintpaul isolates associated with the 2013 outbreak.

The Centers for Disease Control and Prevention estimated that one million foodborne illnesses in the United States are caused by *Salmonella* spp. every year (1). *Salmonella enterica* subsp. *enterica* serovar Saintpaul is particularly robust and able to survive in aquatic environments. Because of its persistence in the environment, *S*. Saintpaul has been involved with multiple fresh produce-borne outbreaks (2). In 2013, the CDC reported an *S*. Saintpaul outbreak in 18 states. Among 84 ill persons for whom information was available, 28% had been hospitalized, and no death was reported (3). The U.S. Food and Drug Administration (FDA) and state investigators traced the origin of the outbreak to contaminated cucumbers from Mexico (3). In this announcement, we report the complete genomes of three *S*. Saintpaul clinical isolates (CFSAN004173, CFSAN004174, and CFSAN004175) associated with the 2013 multistate outbreak linked to cucumbers in the United States. The clinical isolates were collected from stool samples in California and displayed the pulsed-field gel electrophoresis pattern of JN6X01.0161 and JN6A26.0020.

*S*. Saintpaul isolates were cultured in Trypticase soy broth (Becton, Dickinson, Franklin Lakes, NJ, USA) overnight at 37°C. The *S*. Saintpaul genomic DNA were prepared and sequenced based on previously reported procedures (4, 5). The genomes were *de novo* assembled using the PacBio Hierarchical Genome Assembly Process version 3.0 (6). The assembled sequences were annotated using the NCBI Prokaryotic Genome Annotation Pipeline and deposited at DDBJ/EMBL/GenBank.

All three *S*. Saintpaul isolates contain an IncI plasmid. Isolate CFSAN004173 (CDC_2013K-0458) was sequenced with 157× coverage. The genome sizes for the chromosome and plasmid were 4,777,591 bp and 81,408 bp, respectively. A total of 4,896 genes were identified on the chromosome. The G+C content for the chromosome and plasmid were 52.21% and 50.10%, respectively. Isolate CFSAN004174 (CDC_2013K-0457) was sequenced with 140× coverage. The chromosome comprised 4,777,591 bp, contained a total of 4,897 genes, and had a G+C content of 52.21%. The plasmid size was 81,410 bp with a G+C content of 50.10%. Isolate CFSAN004175 (CDC_2013K-0456) was sequenced with 147× coverage. The isolate contained a 4,777,591-bp chromosome and an 81,409-bp plasmid. The complete genome consisted of 4,897 genes with a G+C content of 52.21% for the chromosome and 50.10% for the plasmid.

PHAST analysis identified the presence of prophages Gifsy-1, Entero-ST104, and Burkho-BcepMu in all three *S*. Saintpaul isolates (7).

## Accession number(s).

The complete genome and plasmid sequences of the three *S*. Saintpaul isolates reported here are available in GenBank under the accession numbers CP019172, CP019173, CP019204, CP019205, CP019206, and CP019207.
